# Modeling the Cost-Effectiveness of Adjuvant Osimertinib for Patients with Resected *EGFR*-mutant Non-Small Cell Lung Cancer

**DOI:** 10.1093/oncolo/oyac021

**Published:** 2022-03-14

**Authors:** Christopher A Lemmon, Emily C Zabor, Nathan A Pennell

**Affiliations:** 1 Department of Hematology and Medical Oncology, Cleveland Clinic Taussig Cancer Institute, Cleveland, OH, USA; 2 Department of Quantitative Health Sciences, Cleveland Clinic Lerner Research Institute, Cleveland, OH, USA

**Keywords:** cost-effectiveness, osimertinib, *EGFR*-mutant, non-small cell lung cancer

## Abstract

**Introduction:**

The epidermal growth factor receptor (EGFR) tyrosine kinase inhibitor osimertinib was recently approved for resected *EGFR*-mutant stages IB-IIIA non-small cell lung cancer due to improved disease-free survival (DFS) in this population compared with placebo. This study aimed to evaluate the cost-effectiveness (CE) of this strategy.

**Materials and Methods:**

We constructed a Markov model using post-resection health state transitions with digitized DFS data from the ADAURA trial to compare cost and quality-adjusted life years (QALYs) of 3 years of adjuvant osimertinib versus placebo over a 10-year time horizon. An overall survival (OS) benefit of 5% was assumed. Costs and utility values were derived from Medicare reimbursement data and literature. A CE threshold of 3 times the gross domestic product per capita was used. Sensitivity analyses were performed.

**Results:**

The incremental cost-effectiveness ratio for adjuvant osimertinib was $317 119 per QALY-gained versus placebo. Initial costs of osimertinib are higher in years 1-3. Costs due to progressive disease (PD) are higher in the placebo group through the first 6.5 years. Average pre-PD, post-PD, and total costs were $2388, $379 047, and $502 937, respectively, in the placebo group, and $505 775, $255 638, and $800 697, respectively, in the osimertinib group. Sensitivity analysis of OS gains reaches CE with an hazard ratio (HR) of 0.70-0.75 benefit of osimertinib over placebo. A 50% discount to osimertinib drug cost yielded an ICER of $115 419.

**Conclusions:**

Three-years of adjuvant osimertinib is CE if one is willing to pay $317 119 more per QALY-gained. Considerable OS benefit over placebo or other economic interventions will be needed to reach CE.

## Teaser

The EGFR tyrosine kinase inhibitor osimertinib was recently approved for resected *EGFR*-mutant stage IB-IIIA non-small cell lung cancer due to evidence of improved disease-free survival. This article evaluates the cost effectiveness of adjuvant osimertinib after resection in this population.

**Implications for Practice**

This model analysis demonstrates that adjuvant osimertinib after resection in *EGFR-mutant* non-small cell lung cancer will require an overall survival benefit with a risk reduction of around 25%-30% over placebo to become cost-effective, while drug cost reduction strategies can also help improve this. The data generated in this model serve to better inform clinicians and provide additional insight to help guide patient directed discussions regarding this treatment strategy.

## Introduction

Non-small cell lung cancer continues to be one of the most common and most lethal cancer diagnoses worldwide, with adenocarcinoma accounting for the majority of cases.^1^ Those diagnosed with early or locally advanced non-small cell lung cancer (NSCLC) account for approximately 45% of new cases, with roughly 20%-30% being considered resectable.^2^ Adjuvant chemotherapy is associated with only a modest survival benefit of approximately 5%, and despite the intent for cure, recurrence rates remain high with over half of patients seeing their cancer return by 5 years, leading to poor overall outcomes.^3^

Approximately 15%-20% of patients in the US will harbor an activating mutation in the epidermal growth factor receptor (*EGFR*), with incidence up to 50% in some Asian populations.^4^ The use of targeted treatments for patients whose tumors harbor these mutations has demonstrated significant benefit in recent years in the metastatic NSCLC setting. Osimertinib is a third-generation EGFR tyrosine kinase inhibitor (TKI) approved for use in the first-line metastatic setting in those patients with activating *EGFR* exon 19 deletion, or exon 21 L858R substitution based on demonstrated survival benefits in the FLAURA trial.^5^

Building on this, the phase III ADAURA trial sought to study the benefit in earlier stages.^6^ This study compared the use of 3 years of adjuvant osimertinib versus placebo in those with resectable early-stage NSCLC and was unblinded early after demonstrating a significant benefit in disease-free survival (DFS) with osimertinib when compared with the placebo group [median DFS not reached for osimertinib vs 19.6 months for placebo, (HR 0.17, 95% CI: 0.12, 0.23; <.0001)]. Patients also demonstrated a reduction in distant recurrence with locoregional and distant recurrence rates of 7% and 4%, respectively, in the osimertinib arm, differing from what was seen in the placebo group with 18% of recurrences being locoregional and 28% being distant. Additionally, CNS recurrence was also reduced in the osimertinib arm with 1% having recurrence versus 10% in the placebo arm.^7^ These findings led to accelerated approval from the United States Food and Drug Administration (FDA) in this setting.^8^

These substantial early promising findings have led to debate regarding early clinical implementation due to several factors, including the immaturity of the data, findings from prior targeted EGFR-TKI adjuvant trials that did not establish overall survival (OS) benefit, and the presumed high associated costs of treatment.^9-11^ In this analysis, we evaluate the cost-effectiveness (CE) of 3 years of adjuvant osimertinib in this setting to provide additional insight.

## Materials and Methods

A Markov model was constructed using post-resection health states in a simulated patient population consisting of resected *EGFR*-mutated NSCLC. Health states were categorized as pre- and post-progression and were divided into CNS recurrence-positive and CNS recurrence-negative states.

Disease-free survival data reported from the interim analysis of the ADAURA trial were used.^6^ Kaplan-Meier curves reported in the trial were digitized using previously described methods and then individual patient level data were reconstructed from the curves.^12-16^ We first used Martingale residuals to test whether the proportional hazards assumption held between trial arms and if not, separate models were fit to data from the different arms. Parametric Exponential, Weibull, Gamma, Log-normal, Log-logistic, and Generalized Gamma models were fit to the reconstructed data. The predicted survival curves were compared with the observed survival curves, and the Akaike information criterion (AIC) and Bayesian information criterion (BIC) were used to select the model with the best fit. The proportional hazards assumption was not met for either CNS-positive or CNS-negative disease, so separate models were fit to each arm. The log-normal model had the lowest AIC and BIC in the CNS-positive placebo arm (μ = 1.94, σ = 1.22), placebo arm (μ = 1.11, σ = 1.44), and the CNS-negative osimertinib arm (μ = 2.19, σ = 1.13). Several models appeared to fit the data equally well in the CNS-positive osimertinib arm, so the log-normal model was selected for consistency (μ = 2.66, σ = 0.91). Transition probabilities from post-resection states of no evidence of disease (NED) to disease recurrence or death were generated from these parametric distributions.

The percentage of events that were recurrence versus death were used as the transition probabilities from recurrence or death to progressive disease (PD) in each arm. At the interim analysis of ADAURA, 33/39 (85%) CNS-positive DFS events were recurrence in the placebo arm, 4/6 (67%) CNS-positive DFS events were recurrence in the osimertinib arm, 118/120 (98%) CNS-negative DFS events were recurrence in the placebo arm and 31/31 (100%) CNS-negative DFS events were recurrence in the osimertinib arm.^6^ The transition from NED to death, representing non-disease-related deaths, was generated from WHO mortality data for a male in the US who is 63 years old, the average age of ADAURA participants.

Kaplan-Meier curves of overall survival (OS) from the reference arms of AURA3 and FLAURA were digitized and reconstructed data were obtained as described previously.^5,17^ These data were combined and after fitting parametric models to the combined reconstructed data, the Gamma model had the lowest AIC and ` (α = 1.86, β = 0.56) and was used to generate transition probabilities from PD to death in the CNS-negative and CNS-positive placebo groups. A 5% OS benefit for osimertinib was assumed in the primary analysis, and a range of OS benefit for osimertinib from 0% to 45% was assessed in sensitivity analysis as true OS benefit of osimertinib is currently unknown.

Cost data were collected from Medicare reimbursement data and recent literature.^19-24^ Cost inputs considered included drug costs, clinical management costs, and costs of testing at time of progression, end-of-life costs, as well as generalized costs associated with CNS-positive disease. All costs are in US dollars and were adjusted for inflation (Table 1). Osimertinib treatment was considered for up to 3 years in the adjuvant setting, as per the ADAURA protocol.^6^ All hypothetical patients were assumed to have received equal treatment prior to model randomization. Patients entering the PD state were assumed to be re-treated with osimertinib for up to 2 years for this recurrence based on prior data showing efficacy of this strategy, and based on best estimates of duration of treatment.^5,18^ Additional treatment lines beyond initial recurrence were not considered due to variability of possible treatment decisions in this setting.

**Table 1.** Cost inputs to the Markov model.

Parameter	Value
EGFR testing—one-time cost for all on osi or at progression	$324.58
Osimertinib—per year, up to 3 years or until progression	$222 196
Health care costs in NED—first 3 years	$1078.76
Health care costs in NED—years 4 and 5	$539.38
Health care costs in NED—after year 5	$296.69
Diagnosis of PD—one-time cost	$7202.88
Osimertinib—per year, at PD for the first 2 years	$222 196
Health care costs in PD—annual	$1186.76
Average lifetime CNS+ PD trt/AE—one-time cost	$43 598.83
MRIs in CNS+ PD—annual	$1482.56
Palliative/end of life cancer costs	$78 571.06

Abbreviations: Osi, osimertinib; NED, no evidence of disease; PD, progressive disease; CNS, central nervous system; trt/AE, treatment-related toxicity/adverse event.

Clinical utility states for the pre-specified health states were derived from literature. The quality-adjusted life years (QALYs) for post-resection NED state were 0.83 and 0.812 in the placebo and osimertinib arm, respectively, and the QALYs for CNS-negative and CNS-positive disease were 0.71 and 0.55, respectively.^25-28^ Disutility values were also determined for grade 3 adverse events (Table 2).^29-31^ Probabilities of these adverse effects were derived from the ADAURA and FLAURA trails for pre-progression and post-progression states, respectively.^5,6^ A 3% discount rate per year was considered to costs and a 5% discount per year was considered for utility measurements.^32^ A CE threshold was set at $195 000 as 3 times the US gross domestic product per capita.^33^

**Table 2.** Adverse event probability, disutility, and cost inputs to the Markov model.

Parameter	Probability	Disutility	Cost
Diarrhea—NED osi arm	^.^023	–0.32	$159.68
Rash/stomatitis—NED osi arm	^.^018	–0.15	$169.97
Decreased appetite- NED osi arm	^.^005	–0.39	$38.25
Pneumonia/sepsis—NED osi arm	^.^015	–0.50	$42 928.16
Diarrhea—PD osi arm	^.^025	–0.32	$159.68
Rash/itching/dry skin—PD osi arm	^.^018	–0.15	$169.97
Decreased appetite—PD osi arm	^.^011	–0.39	$38.25
Pneumonia/sepsis—PD osi arm	^.^047	–0.50	$42 928.16
Pneumonities/respiratory—PD osi arm	^.^05	–0.40	$16 584.44
Neurocognitive defects—CNS+	^.^10	–0.35	—^a^
Radionecrosis—CNS+	^.^01	–0.50	—^a^

Incorporated into costs of average lifetime CNS+ PD trt/AE.

Abbreviations: Osi, osimertinib; NED, no evidence of disease; PD, progressive disease; CNS, central nervous system; trt/AE, treatment-related toxicity/adverse event.

The primary endpoint for this study was the incremental CE ratio (ICER) stated as cost per QALY-gained of osimertinib when compared with placebo. Additional endpoints were the average pre- and post-progression costs as well as total costs of the osimertinib strategy versus placebo.

Both deterministic and probabilistic sensitivity analyses were conducted to assess the sensitivity of the model to the various inputs. Deterministic sensitivity analyses included the incremental increase in potential OS benefit in terms of reduced risk of death, and drug annual cost discount percentage among others. Probabilistic sensitivity analyses placed distributions around each of the model inputs defined in the Markov model. Then, the Markov model was re-run 1000 times with model inputs resampled from the specified distributions each time, to obtain a range of possible model results, representing the uncertainty in the model. All statistical analyses were conducted in R software version 4.0.3 (R Core Development Team, Vienna, Austria) including the “heemod” package.^34^

This research did not receive any specific grant from funding agencies in the public, commercial, or not-for-profit sectors.

## Results

The average pre-PD, post-PD, and total costs were $2388, $379 047, and $502 937, respectively, in the placebo group, compared with $505 775, $255 638, and $800 697, respectively, in the osimertinib group. Average QALYs in the osimertinib group were 5.108 compared with 4.169 in the placebo group. The average total cost difference for osimertinib compared with placebo, assuming an improvement in OS of 5%, was $297 759.90 and the QALY difference was 0.9389, yielding and ICER value of $317 119.90 for osimertinib with reference to placebo.

**Figure 1.**
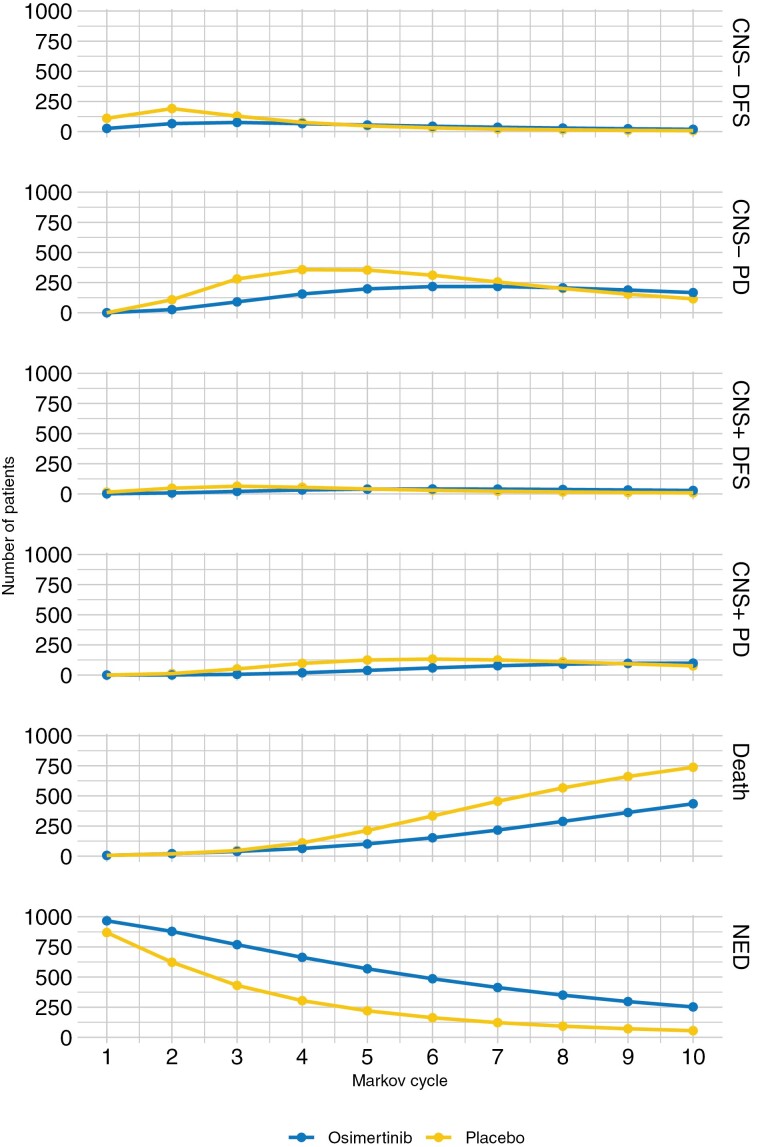
Health state transitions over time.

Heath state transitions show placebo patients reached the PD state quicker than those in the osimertinib group. Placebo group patients also had a higher incidence of death than the osimertinib group starting around year 3. More patients in the osimertinib arm remained in the disease-free state throughout the 10-year time course. In years 8-10 of the simulation, the osimertinib arm achieves higher rates of progression than placebo, signifying delayed entry into this state with osimertinib (Fig. 1).

**Figure 2.**
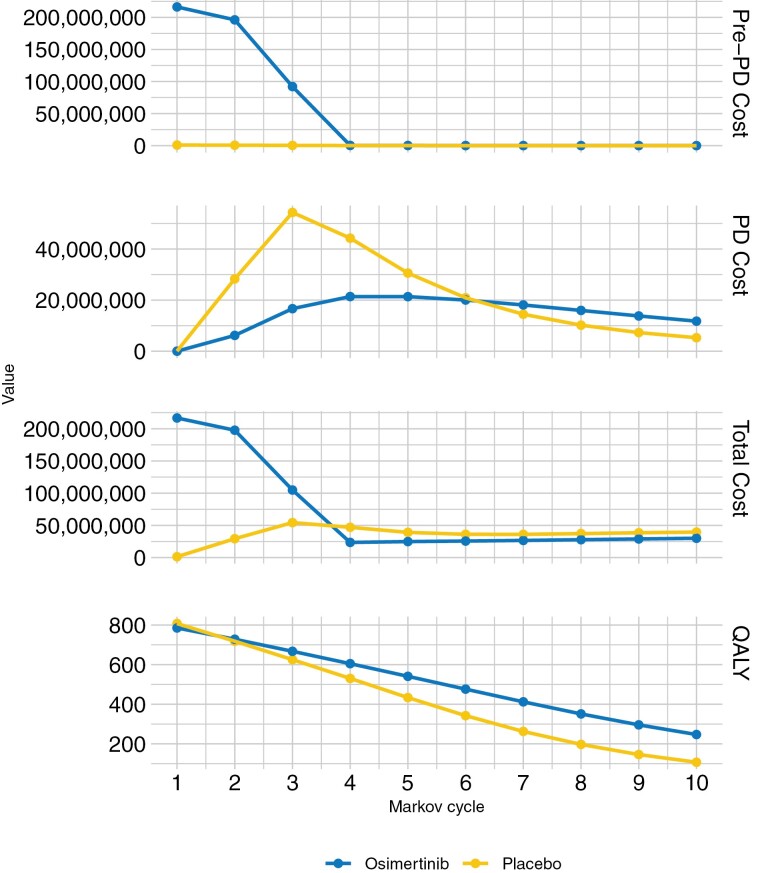
Costs and quality-adjusted life years over time.

Initial costs in the osimertinib arm are higher than those in the placebo arm through the first 3 years with much of the cost accumulating as drug expense. The osimertinib arm costs become lower than the placebo group in year 4 onward, with similar costs after year 7. Costs due to PD are higher in the placebo group through the first 6.5 years. QALYs were higher in the osimertinib arm throughout the 10-year time course (Fig. 2).

**Figure 3.**
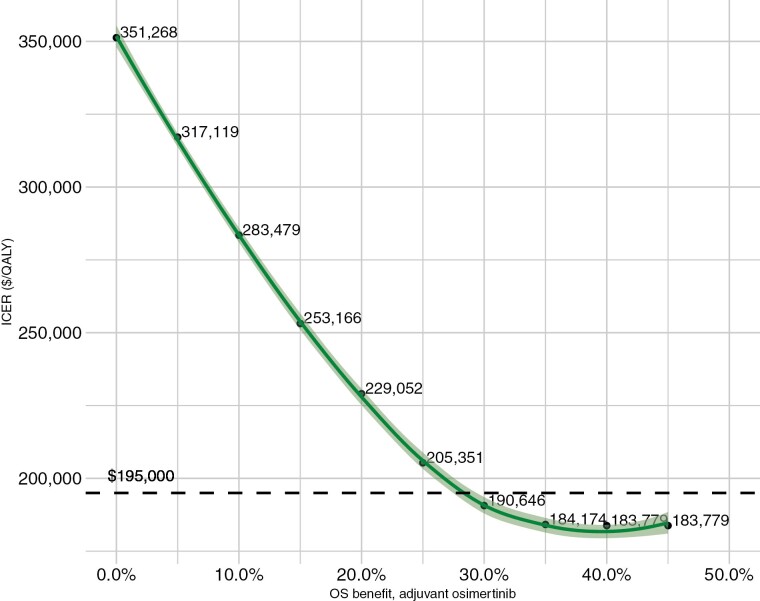
Sensitivity analysis of incremental overall survival benefit of osimertinib over placebo.

Deterministic sensitivity analyses of incremental increases in OS benefit were performed demonstrating progressive improvements in the base-case ICER, and reaching the CE threshold of $195 000 between a survival benefit risk reduction of 25%-30% for osimertinib over placebo (HR 0.75-0.70; Fig. 3).

Evaluation of the effect of drug discount of the annual cost of osimertinib was also performed with assessment of 10%, 25%, and 50% annual cost discounts. This led to reductions in the ICER value to $276 779, $216 269, and $115 419, respectively; reductions of 13%, 32%, and 64%, respectively, compared with the base-case ICER.

**Figure 4.**
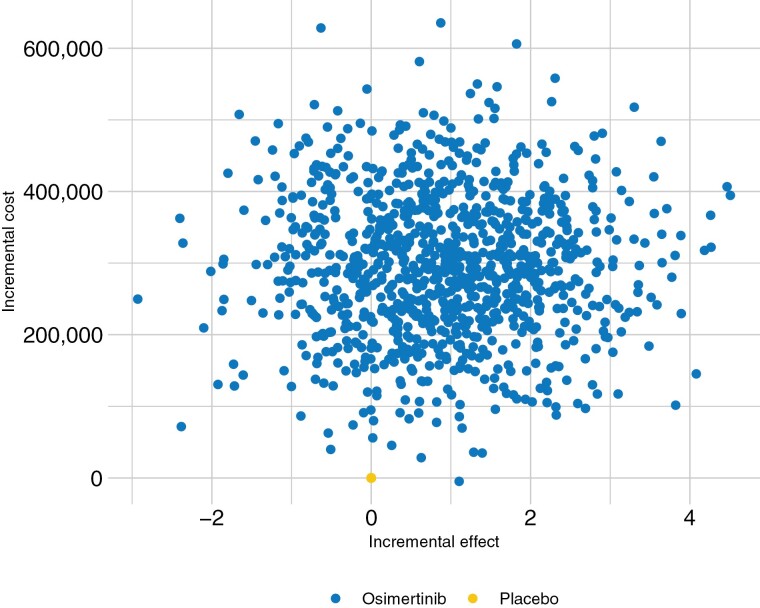
Uncertainty of the incremental cost and effect of osimertinib on the cost-effectiveness plane, with placebo as the reference.

Probabilistic sensitivity analysis results revealed a variety of possible model results were the input parameters to vary, centered around our model’s ICER of $317 119.90 (Fig. 4).

## Discussion

Osimertinib was approved by the FDA for use as adjuvant therapy for *EGFR*-mutant early-stage NSCLC after the ADAURA trial demonstrated early DFS benefit in this population.^6,8^ However, there has been substantial debate regarding clinical implementation of this strategy due to a variety of issues, including the choice of DFS as the trial’s primary endpoint as prior trials evaluating the use of earlier generation EGFR-TKI in the adjuvant setting have not demonstrated OS benefit.^9-11,35,36^ This, as well as the overall immaturity of both the DFS and OS data, and the high associated drug cost of treatment, has raised the question of should clinicians incorporate this strategy into practice now, or await more concrete survival data? The ADAURA OS data will likely not be mature and available for several years. Additionally, if the benefit is large enough, is it worthwhile to delay recurrence regardless of OS benefit?

To our knowledge, this is the first CE evaluation of osimertinib in the adjuvant setting for early-stage NSCLC. It is also unique in its design, using sensitivity analyses in OS for input to predict potential CE as current true trial data remains immature. Prior models have evaluated the CE of osimertinib in the metastatic setting compared with chemotherapy, and compared with other earlier-generation EGFR TKIs.^37,38^ These found that osimertinib was not cost-effective in the first-line metastatic setting, primarily due to drug acquisition cost, despite OS benefits.

Similarly, our model demonstrated that at the current cost and with an assumed improvement in OS of 5% over placebo, osimertinib would only be cost-effective if one was willing to pay $317 119 per QALY-gained, well above the pre-specified threshold of $195 000. However, in sensitivity analysis, as the potential benefit in OS of osimertinib increases, the ICER progressively declines and eventually reaches the threshold of CE with a risk reduction for death between 25% and 30% benefit (HR for OS of 0.75-0.70).

This raises one of the primary questions surrounding the ADAURA trial results. Is the significant DFS benefit reported sufficient to incorporate this strategy into clinical practice without mature long-term survival data? Prior randomized studies of adjuvant EGFR-TKIs established DFS benefit, but these have not translated to an improvement in OS. In EVAN, 2 years of Erlotinib in Chinese patients with stage IIIA *EGFR*-mutant NSCLC demonstrated 2- and 3-year DFS of 81.4% and 54.2%, respectively, compared with 44.6% and 34.4% for chemotherapy.^35^ Updated analysis for OS demonstrated a median OS of 84.2 months in the erlotinib arm versus 61.1 months in the chemotherapy arm with an HR of 0.318; however, the limited patient population and lack of statistical power limit the interpretation of these results.^39^ Similarly, ADJUVANT CTONG1104 study of Chinese patients with stage II or IIIA *EGFR*-mutant NSCLC compared 2 years of Gefitinib versus chemotherapy and yielded median DFS benefits of 28.7 months with TKI therapy versus 18 months with chemotherapy, but the benefit was lost by year 3 and there was no benefit in OS.^36^

This may lead to other questions as well. If patients begin to recur after 3 years, should patients continue treatment for a longer duration, or indefinitely? In the SELECT trial, it was noted that patients began recurring after therapy completion, indicating a possible suppressive effect of therapy in those that would have had persistent disease after resection, rather than a curative effect.^18^ While there appears to be no significant difference in the health-related quality of life data for patients on osimertinib compared with placebo, the financial cost of indefinite therapy would be considerable.^40^ This becomes especially important in the group of resected patients that would have ultimately been “cured” without additional EGFR-targeted treatment, who would be subjected to these costs and potential drug toxicity without any benefit. Establishing a biomarker for those that would benefit from additional therapy will be an important research prerogative.

Additionally, while considering the different aspects of toxicity, consideration of several patient aspects is also needed. Distant and CNS recurrences can come with significant morbidity, and treatment of these can drive up costs quickly.^41^ Reducing the rates of these as was seen in ADAURA in favor of locoregional recurrences could potentially improve patient quality of life, and reduce long-term costs. Additionally, psychologic toxicity could also be a factor, as it may provide relief for patients to know they are disease-free.^42^ A prolonged delay in time to recurrence could provide these patients with more time for new drug trials or approvals that may benefit them that they may not have had access to had they recurred or died sooner.

Lastly, financial factors need to be considered. Similar to the findings in the metastatic setting, the drug cost of osimertinib limits its CE and even with substantial OS benefits may not reach CE, especially in countries with more limited health-care expenditure. In the US, this treatment is likely to be implemented broadly regardless of CE, as has been done in the metastatic setting. The US pays more for cancer drugs than most other countries and a substantial portion of this cost unfortunately often falls to the patients.^43^ Discounts to drug cost can have a substantial impact on the overall CE of this treatment, as is demonstrated in our analysis with discounts having significant impacts on the ICER. However, depending on a patient’s health insurance, the financial toxicity for which the patient is still responsible may be excessive and can cause substantial distress or even prohibit treatment, regardless of whether a pre-specified CE threshold is met. While systemic legislative reform of drug pricing is needed, this may be unlikely given the need for political intervention. However, patient assistance programs are often available through pharmaceutical companies, and other financial grants can help to reduce the burden to patients. There is a continued need to explore ways to reduce the costs of these drugs, ensure meaningful clinical benefit, and provide honest financial consideration to patients.^44,45^

Our study is limited by the use of assumptions and uncertainty due to modeling that may not accurately reflect real-world scenarios. The inputs and limited health state transitions also limit the simulated patient population’s clinical presentations and overall course of progress. Treatment decisions were limited in the progressed disease state after osimertinib re-treatment due to variation in clinical practice, limiting the real-world applicability once patients enter this state. Treatment duration and survival at recurrence are also best estimates from available data and may not reflect real-world outcomes. Additionally, DFS data were digitized and reconstructed to best fit, and the data from the ADAURA trial are still immature, with many patients enrolled remaining on treatment. While OS is also immature, this was evaluated by 1-way deterministic sensitivity analysis. True survival representations will require awaiting further analysis of the ADAURA trial.

## Conclusion

Our model demonstrates that there is significant cost associated with the use of 3 years of adjuvant osimertinib, with the majority due to associated drug cost, limiting the CE of this strategy. However, a hypothetical OS benefit of osimertinib of 25%-30% over placebo would improve the ICER to meet a prespecified CE threshold of $195 000. Additional studies of osimertinib after concurrent chemoradiation in those with locally advanced unresectable disease (LAURA trial, NCT03521154) will also provide further insight and a study of neoadjuvant osimertinib (Neo-ADAURA trial, NCT04351555) may limit the need for adjuvant treatment and reduce the risk of overtreating. Ultimately, mature OS data from ADAURA are needed for true CE measure, and additional considerations involving the financial burden to the patient and the utility in delaying recurrence will need to be further elucidated.

## Data Availability

The data underlying this article are available in the article.
